# Towards an extremely high resolution broad-band flat-field spectrometer in the ‘water window’^1^

**DOI:** 10.1107/S1600577519004648

**Published:** 2019-06-04

**Authors:** Zhuo Li, Bin Li

**Affiliations:** a Shanghai Institute of Applied Physics, Chinese Academy of Sciences, Jiading District, Shanghai 201800, People’s Republic of China; b Shanghai Synchrotron Radiation Facility, Shanghai Advanced Research Institute, Zhangjiang Laboratory, Chinese Academy of Sciences, Pudong District, Shanghai 201204, People’s Republic of China; c School of Physical Science and Technology, ShanghaiTech University, Shanghai 201210, People’s Republic of China; d University of Chinese Academy of Sciences, No. 19(A) Yuquan Road, Shijingshan District, Beijing 100049, People’s Republic of China

**Keywords:** spectrometer, X-ray optics, resolution enhancement, geometric optics, ray tracing, diffraction principle, optical aberration analysis and optimization

## Abstract

The optical design of a novel spectrometer is presented, combining a cylindrically convex pre-mirror with a cylindrically concave varied-line-spacing grating (both in the meridional) to deliver a resolving power of 100000–200000 in the ‘water window’ (2–5 nm). Most remarkably, the extremely high spectral resolution is achieved for an effective meridional source size of 50 µm (r.m.s.); this property could potentially be applied to diagnose SASE-FEL and well resolve individual single spikes in its radiation spectrum. The overall optical aberrations of the system are well analysed and compensated, providing an excellent flat-field at the detector domain throughout the whole spectral range. Also, a machine-learning scheme – SVM – is introduced to explore and reconstruct the optimal system with high efficiency.

## Abstract 2

An original and novel design scheme has been formulated to achieve an extremely high resolving power for a broad-band X-ray spectrometer with a relatively large source size, implementing a meridional pre-convex mirror to enhance the resolving power substantially while maintaining minimal intrinsic optical aberrations for the whole system to cast a decent flat-field at the detector domain throughout the spectral range.

## 1. Introduction   

In the past few decades, X-ray spectrometers have accomplished rapid development driven by advanced light sources such as synchrotron radiation facilities and free-electron lasers (FELs), and have been widely used for exploring various intriguing research topics especially in the extreme ultraviolet or soft X-ray regimes, *e.g.* applications of tokamak plasmas and magnetic confinement fusion (Schwob *et al.*, 1987 ▸), laser-produced warm dense matter and extreme energy density states (Schwanda *et al.*, 1993 ▸), stellar or planetary interior properties (Xiong *et al.*, 2011 ▸), instrument development and applications for advanced light sources (Koike *et al.*, 2003 ▸). The technique is necessary for providing high spectroscopic resolution in physical, chemical, photonic and biological research. Pursuing better spectral resolution always remains a strong motivation for researchers, helping them to envision subtler details in materials, and explore previously unobserved phenomena. The ‘water window’, spanning the wavelength range 2–5 nm, is able to provide excellent contrast imaging for C or O atoms and related structures; this outstanding property could be utilized to image and analyze biological cells or microstructures *in vitro* and potentially *in vivo*. ‘Water window’ spectroscopy is also a novel probe for material properties and electron energy states.

Previously, high-resolution spectrometers in this spectral range have included the following designs: grating on Rowland circle structure (Namioka, 1959 ▸); single-plane grating grooved in varied line spacing (VLS) (Fan *et al.*, 1992 ▸; Xiong *et al.*, 2011 ▸); single concave VLS grating (Harada & Kita, 1980 ▸; Nakano *et al.*, 1984 ▸); concave mirror pre-focusing the incident beam upstream of a plane grating, creating a real secondary source (Choi *et al.*, 1997 ▸); beam pre-focused by a spherical mirror to converge beyond the VLS grating, creating a virtual source, *i.e.* Hettrick–Underwood design (Hettrick *et al.*, 1988 ▸), which exists in different versions: *e.g.* (i) Hague *et al.* (2005 ▸) employed a Kirkpatrick–Baez (KB) mirror for pre-focusing to correct for spectral astigmatism; (ii) Tondello (1979 ▸) replaced the KB mirror with a toroidal mirror; (iii) Dvorak *et al.* (2016 ▸) added a deflection mirror downstream of the grating to level the outgoing beam; (iv) the Hettrick–Underwood scheme implements a Wolter-type focusing system (Warwick *et al.*, 2014 ▸), *etc*. Beside these, Y. D. Chuang and Y. C. Shao have designed a modular spectrometer whose modules can be conveniently adapted to various research requirements (Chuang *et al.*, 2017 ▸).

In the past, convex mirrors were rarely used. Only the Wolter III focusing system consisting of a hyperbolically convex mirror and an elliptically concave mirror has been adopted in X-ray imaging and microscopy (Wolter, 1952 ▸), where the incoming beam is grazing incident on the convex mirror and the reflected beam is diverging. Its reverse extension lines are converged at one focus of the concave elliptical mirror; the reflected beam from the ellipse is propagating backward and then focused on the other focus. Except for a few reports (Saha, 1985 ▸, 1988 ▸), the characteristics of the Wolter III mirrors have been rarely studied, resulting in a lack of deep and clear understanding. Inspired by the Wolter configuration and based on this previous work, we formulated a delicate high-resolution flat-field spectrometer design for the ‘water window’, combining an upstream pre-divergent convex mirror and a downstream concave VLS grating, which is demonstrated to enhance the resolving power considerably while maintaining a decent flat-field condition throughout the whole spectral range.

Resonant inelastic X-ray scattering (RIXS) spectrometers usually have a very high resolving power, benefiting from an excellent upstream monochromator system, via confining or focusing the beam width down to a few micrometres, producing a small secondary light source for the spectrometer downsteam (Dvorak *et al.*, 2016 ▸). Its detection arm can scan a wide angular range corresponding to various momentum transfers in between the photon and the sample materials. RIXS can be implemented to investigate the energy, momentum and polarization dependence of photon–matter interactions or scattering processes, and hence to reflect the intrinsic properties of charge, spin, orbital, lattice excitation *etc.* (Ament *et al.*, 2011 ▸). With improvements in resolving power, for instance, charge transfer and *d*–*d* excitations (Kuiper *et al.*, 1998 ▸; Harada *et al.*, 2000 ▸), spin excitations in cuprates (Braicovich *et al.*, 2010 ▸; Guarise *et al.*, 2010 ▸) and iron pnictides (Zhou *et al.*, 2013 ▸), high-energy phonons (Braicovich *et al.*, 2010 ▸) and vibrations in single molecules (Hennies *et al.*, 2010 ▸; Pietzsch *et al.*, 2011 ▸) could be thoroughly investigated.

Our efforts are completely different, aiming to achieve such a high resolving power by utilizing a scheme similar to Wolter configurations, *i.e.* inserting a convex mirror upstream of the concave VLS grating. Then, the intrinsic optical nature of the system, and the primary factors influencing the spectral distribution quality and resolution are explicitly analysed to exploit its best performance. This kind of spectrometer can be used to diagnose the radiation properties of FELs, especially for the self-amplied spontaneous emission (SASE) mode. In a SASE process, radiation gain and saturation originate from small random phase noise, and mutual interaction in between the electron bunch and the radiation. The resulting radiation is closely correlated to the electron bunch properties, *e.g.* bunch charge peak current, beam longitudinal and transverse profiles, beam emittance, electron kinetic energy and spread, *etc*. Typically, a saturated FEL radiation pulse possesses high transverse coherence and partial longitudinal coherence, where the pulse’s longitudinal profile in the time domain includes multiple individual coherent spikes which are mutually uncorrelated and incoherent. However, it is extremely difficult to directly measure the temporal profile of a SASE pulse precisely, thus a high-resolution spectrometer could alternatively be used to measure the corresponding spectrum of the SASE pulse. For example, for the FEL radiation in the photon energy range of the ‘water window’ (280–600 eV) up to 1 keV the entire SASE bandwidth (Δ*E*/*E*) is about 1/1000–1/200, while the bandwidth for a typical coherent spike in a SASE pulse is an order less, spanning only 1/20000–1/5000. Resolving well a single spike can not only provide the detailed structures in the SASE radiation spectral domain but also reflect the minimal SASE pulse length in the time domain simutaneously (since Heisenberg’s uncertainty law or transform limit implicates that the SASE pulse length should not be shorter than the reciprocal of the spectral bandwidth of an individual coherence spike in its spectrum) (Engel *et al.*, 2016 ▸). So, the current spectrometer design, providing a very high spectral resolving power of 100000–200000, could determine critical parameters of FEL radiation. In particular, we achieve an extreme spectral resolution for a relatively large source size (50 µm r.m.s.); this exceptional property could enhance the spectral intensity and detection efficiency substantially, which exhibits a promising photon diagnostic scheme for a FEL light source.

The manuscript is organized as follows:

(*a*) The second section[Sec sec2] presents a numerical simulation and algorithm to prove that the convex pre-mirror is a good choice for enhancing the resolving power of a spectrometer. Besides the resolution enhancement, a decent flat-field could be achieved at the detector, since the optical aberrations of the convex mirror propagate downstream to compensate those of the concave grating, thus optimizing the primary aberrations of the overall system.

(*b*) The third section[Sec sec3] explicitly discusses the optimization algorithm, where the machine-learning tool Support Vector Machine (SVM) is introduced and implemented to achieve a set of optimal parameters in the spectrometer design, while the quality evaluation parameter for the spectral imaging is well defined and discussed.

(*c*) The fourth section[Sec sec4] mainly discusses the key parameters of the system (*e.g.* source size, optical aberrations, fabrication errors, *etc.*) determining the ultimate spectral resolution, which is verified by ray tracing. In particular, critical requirements for the slope errors of the optical elements in the high-resolution spectrometer are analysed.

(*d*) Finally, we make a more general and summarizing remark regarding our design, and discuss potential future research and development.

## 2. Numerical simulation   

First, we list a set of parameters fixed in the simulation and discussion throughout this article: (i) light source intensity distribution: Gaussian profile; (ii) size of light source: σ_s_ = 50 µm (r.m.s); (iii) divergence angle of light source: 20 µrad (r.m.s); (iv) grooved density of VLS grating at the centre: *D*_0_ = 24000 lines cm^−1^; (v) grating diffraction order: *m* = 1; (vi) wavelength range: 2–5 nm (water window); (vii) distance from original light source to grating: *L* = 30 m. Here, we are mainly concerned with the beam properties in its meridional coordinate, thus cylindrical substrates (tangentially convex or concave profiles) are adopted for all the optical elements in the system. This is sensible, since the beam divergence of synchrotron radiation or free-electron lasers is quite small; a freely propagating beam in sagittal coordinates would not lead to a large footprint in that direction.

### 2.1. Four types of spectrometer   

**Figure 1:**
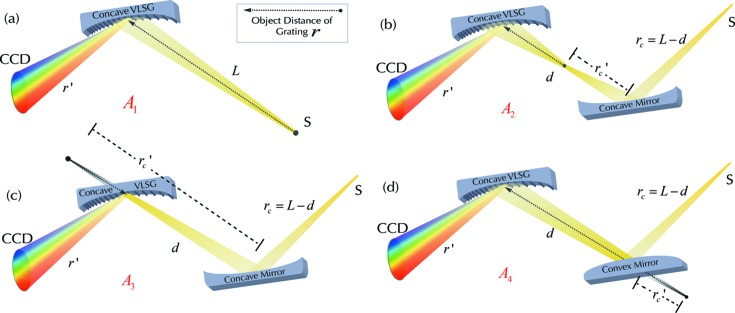
Schematic diagram of four kinds of spectrometer design implementing a concave VLS grating with or without pre-mirror (corresponding to *A*_1_–*A*_4_). *S* represents the light source, *L* is the distance from the original light source to the grating, *d* is the distance between the pre-concave (or convex) mirror and the grating, *r*_c_ and 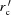 are the object and image distances of the concave (or convex) mirror, respectively, *r* is the object distance of grating indicated by a dotted arrow, *r*′ is the image distance of grating. (*a*) Single concave VLS grating, where the object distance of grating is *r* = *L*. (*b*) The concave VLS grating is combined with a pre-focusing concave mirror, forming a real source for the grating, *r* = 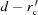 > 0. (*c*) A similar case to (*b*), where the pre-concave mirror forms a virtual source for the grating, *r* = 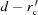 < 0. (*d*) The concave VLS grating is combined with a pre-diverged convex mirror, where the source of the grating is real, *i.e.* *r* = 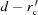 > *d* > 0, since 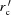 < 0.

The single concave VLS grating spectrometer is shown in Fig. 1(*a*) ▸, and its ideal resolving power is given by (Li & Li, 2018 ▸)
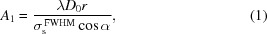
where λ is the wavelength, *r* is the object distance of the grating, *D*_0_ (grating groove density) has been defined previously, 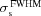 is the original source size in FWHM (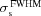 ≃ 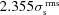), and α is the incident angle of the grating. Since *L* is the distance from the original light source to the grating, then *r* = *L* for this case (denoted by the dotted line arrow). According to equation (1), the resolving power is proportional to the wavelength, the groove density of the grating, the grating object distance *r* (or *L*), inversely proportional to the light source size, and prefers a larger incident angle (or a smaller grazing incidence angle).

As shown in Fig. 1(*b*) ▸, the concave VLS grating is combined with a pre-focusing concave mirror, forming a real secondary source for the grating, *i.e.* the meridional beam focuses upstream of the grating and illuminates it. So, the resolving power is calculated by
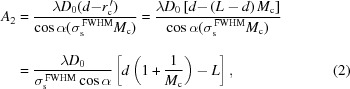
where *r*_c_ and 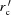 are the object and image distances of the pre-focusing mirror, whose magnification is denoted by 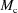 = 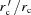 (since 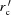 > 0 for this case), and *d* is the separation in between the concave mirror and the grating. So, the object distance of the concave mirror is *r*_c_ = *L* − *d*, the grating object distance can be expressed as 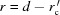 > 0, and the effective source size of the grating is 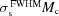.

Fig. 1(*c*) ▸ presents a similar configuration to Fig. 1(*b*) ▸, while the pre-concave mirror forms a virtual source for the grating, *i.e.* the meridional beam focuses behind the grating. This recalls the typical Hettrick–Underwood scheme, associated with a resolving power of
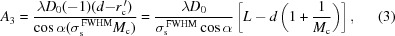
where *M*_c_ > 0 (since 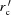 > *d* > 0), the (−1) term in the numerator indicates the virtual source for the grating, and its object distance is 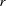 = 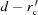 < 0 (virtual source). The rest of the variables in equation (3) are defined in a similar way as for equation (2).

Finally, in Fig. 1(*d*) ▸ the VLS grating is combined with a pre-convex mirror. The incident beam is diverged meridionally by the cylindrical convex mirror, and the virtual image of the convex mirror represents the real source of the grating effectively. The resolving power of the system is
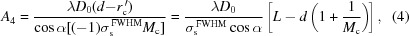
where *M*_c_ < 0 since the pre-convex mirror generates a virtual image (the image distance 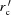 < 0) and the object distance of the grating 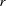 = 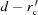 > *d* > 0. Similarly, the (−1) term in the denominator of equation (4) is due to the virtual image of the convex mirror.

**Figure 2:**
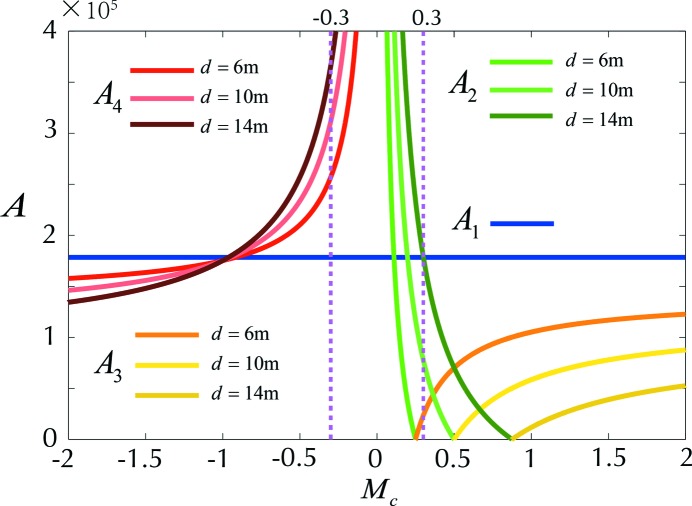
Demonstration of the various resolving power dependences *A*_1_–*A*_4_ on the magnification *M*_c_ provided by the pre-mirror, at three typical spacings *d* (distance in between the pre-mirror and the grating); each of *A*_1_–*A*_4_ are grouped into similar colours. The common parameters for each case are: *L* = 30 m, λ = 5 nm, σ_s_ = 50 µm (r.m.s), α = 89°, *D*_0_ = 24000 lines cm^−1^. *A*_1_ (blue) is constant at about 170000; *A*_2_ (green) would enhance the resolution only when *M*_c_ < 0.3 (with respected to *A*_1_); *A*_3_ (yellow) cannot improve the resolution for any possible value of *M*_c_; *A*_4_ (red) can enhance the resolution as long as |*M*_c_| < 1, where the region −0.3 < *M*_c_ < 0 (or 0 < *M*_c_ < 0.3 for *A*_2_) within the two purple dashed lines corresponds to extremely high resolution. However, the optical aberration for the pre-diverging (focusing) mirror could be unacceptably high. *A*_4_ is a preferable solution to enhance the resolving power when |*M*_c_| < 1 but not too small.

In order to evaluate the performance of these four systems, their resolving powers [refer to equations (1)–(4)] are plotted against *M*_c_ in Fig. 2 ▸, for a set of three different optical element spacings, *d =* 6, 10 and 14 m. Again, the pre-set parameters at the beginning of Section 2 are used for the calculation, *e.g.* *L* = 30 m, σ_s_ = 50 µm (r.m.s), α = 89°, *D*_0_ = 24000 lines cm^−1^. Since the resolving power is wavelength dependent, here only the results for λ = 5 nm are presented.

In Fig. 2 ▸, *A*_1_ (blue) is the baseline case and has a constant resolution of ∼170000, where *d* or *M*_c_ are not applicable since only a single concave grating is used in the system. For the other three configurations, only if the values of *A*_2_–*A*_4_ are greater than *A*_1_ is the resolving power ‘enhanced’. For *A*_2_ (three green curves crossing the centre of the graph vertically, with only minor differences in colour), the resolving powers monotonously decrease with *M*_c_ for each *d*, only if *M*_c_ is less than 0.304 (for *d* = 14 m), the resolution would be greater than *A*_1_ (while for *d* = 10 m, *M*_c_ < 0.200; for *d* = 6 m, *M*_c_ < 0.111). On the other hand, with *M*_c_ increasing, the focus of the pre-focusing mirror will gradually move to the surface of the grating; in that circumstance the resolving power declines down to zero. Further increasing *M*_c_, the system will transit to *A*_3_, *i.e.* the Hettrick–Underwood design (yellow curves in bottom-right corner), where the focal spot behind the grating corresponds to a virtual source of the grating. *A*_3_ monotonously increases with *M*_c_ for all *d* values, and an apparently smaller *d* is associated with a relatively higher resolving power. However, since *A*_3_ is always less than *A*_1_ for any case, *A*_3_ is unable to enhance the resolving power. For *A*_4_ (three red curves in top-left corner), the pre-convex mirror generates a virtual image, *i.e.* 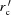 < 0 and *M*_c_ < 0. It is observed that *A*_4_ monotonously decreases with |*M*_c_| for all *d* values. When |*M*_c_| < 1, the resolving power would be enhanced (*A*_4_ > *A*_1_). Especially when |*M*_c_| becomes smaller than 0.3 (the region confined by vertical dashed lines), *A*_4_ gains significantly (similar as *A*_2_). But it needs to be pointed out that a too small value of |*M*_c_| is generally associated with unacceptably large optical aberrations delivered by the pre-focusing (*A*_2_) or diverging (*A*_4_) mirrors, and should be avoided in the system design. According to Fig. 2 ▸ and the discussion above, *A*_2_ can only achieve resolving power enhancement within the region |*M*_c_| < 0.3, while *A*_4_ could achieve this outside the region, having a larger flexibility for the system design. Therefore, configuration *A*_4_ with a pre-convex mirror was chosen to develop an optimal spectrometer with enhanced resolving power (with respect to *A*_1_).

### 2.2. Resolution enhanced flat-field spectrometer   

We need to proceed in the following steps to design an enhanced flat-field spectrometer, using configuration *A*_4_.

(*a*) Establish a set of fundamental parameters (refer to the beginning of Section 2). Gaussian-light source; source size: σ_s_ = 50 µm (r.m.s); source divergence angle: 20 µrad (r.m.s); *D*_0_ = 24000 lines cm^−1^; *m* = 1; wavelength range: 2–5 nm; *L* = 30 m; and optical elements with meridionally cylindrical profiles.

(*b*) Determine the image distance of grating *r*′. The magnification of a diffraction grating is
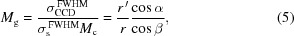
where the minimum value of 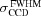 is set to the pixel size of the CCD, which is the spatial limit to resolve the spectral distribution at the detector; 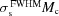 represents the effective source size of the grating created by the pre-convex mirror; α and β are the incidence and diffraction angles of the grating, respectively.

From the previous discussion, the object distance of the grating is *r* = *d* − (*L* − *d*)*M*_c_ (where −1 < *M*_c_ < 0 for configuration *A*_4_ in Fig. 2 ▸). Then the image distance of the grating should meet the following requirement,
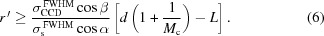
So, *r*′ is a function of *d* and *M*_c_, and could be interpreted as follows: an upstream pre-convex mirror creates a new light source with a new effective object distance for the grating, which determines the minimal image distance the grating should have.

(*c*) Achieve the ‘flat field’. The groove density of a VLS grating is
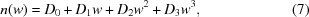
where the VLS coefficients *D*_*i*_ could be optimized through the elimination of optical aberrations in various orders for the system, using the scheme we developed previously (Li & Li, 2018 ▸). In addition, the grating on a cylindrically concave substrate with optimized VLS coefficients allows the achievement of an excellent meridional ‘flat-field’ at its detector plane.

According to Fermat’s principle for geometrical optics, the optimal imaging in meridional coordinates could be achieved through zeroing the first-order derivative of the light-path function connecting the light source and the image via optics (since the grating is a dispersive optic, various wavelengths are associated with different preferable optical paths) (Samson *et al.*, 1998 ▸). In particular, the *F* terms, *e.g.* the first few dominants, should satisfy the following equations crossing the wavelength range,
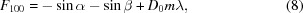


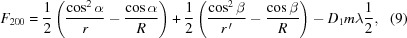


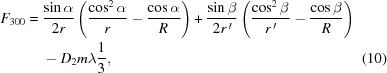


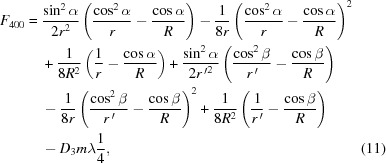
where *R* is the cylindrical radius of the grating. More specifically, the equation of *F*_100_ is actually the grating formula; *F*_200_ is related to the meridional focus, and could be utilized to characterize the ‘defocus’ over the whole spectral range; and *F*_300_ and *F*_400_ are associated with the ‘coma’ and ‘spherical aberration’, respectively.

The imaging distance of the grating which achieves the optimal flat-field for the entire spectral range, according to (Li & Li, 2018 ▸)
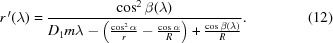
Each set of parameters would lead to a unique optimal meridional radius *R* and coefficient *D*_1_ only, then *D*_2_ and *D*_3_ could be derived at the central wavelength λ_0_ by letting *F*_300_(λ_0_) = 0 and *F*_400_(λ_0_) = 0 via equations (10) and (11).

(*d*) Correction of aberrations. The above discussion is only applicable to a single concave grating. In the case where a pre-focusing (divergent) mirror is implemented in the system, the optical aberrations propagation from the upstream mirror need to be taken into account.

The primary aberrations of an upstream convex mirror could be calculated in a similar way as equations (9)–(11), using the optical path function and the relevant *F*-terms,
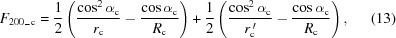


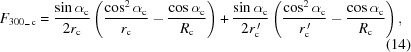


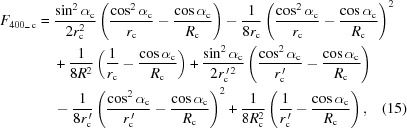
where the reflection angle from the convex mirror is equal to the incident angle α_c_, *r*_c_ and 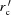 are the object and image distance of the convex mirror, respectively, and *R*_c_ is its meridional radius.

Setting *F*_200_c_ = 0 leads to
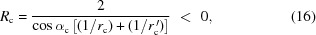
since *r*_c_ = *L* − *d* and 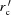 = (*L* − *d*)*M*_c_ (−1 < *M*_c_ < 0), where the convex mirror forms a reduced virtual image. So, the overall *F* terms for the system consisting of a pre-convex mirror and a concave VLS grating could be recalculated by
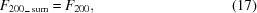


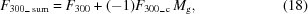


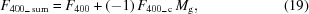
where *M*_g_ is the magnification of the grating [refer to equation (5)], since the *F* term is proportional to the line width of the spectrum; the (−1) term in the formula is due to the virtual image created by the convex mirror (while it represents the real source of the grating effectively).

When the beam passes through the optical system, the optical aberrations will broaden the beam size from the ideal spectral imaging distribution, the aberration broadening effect in the detector domain could be expressed as
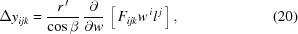
where *w* is the illuminated meridional length of the grating, *l* is the illuminated sagittal length, and *F*_*ijk*_ defines the optical aberrations in various orders, *e.g.* in equations (17)–(19) (the subscript *i* or *j* denotes the meridional or sagittal coordinate, respectively, *k* represents the orthogonal coordinate with *i* and *j*).

Therefore, the meridional radius *R* and coefficient *D*_1_ of the VLS grating could be re-optimized by letting *r* = *d* − 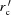 > 0 in equation (12) to obtain the best flat-field for the whole spectral range, while *D*_2_ and *D*_3_ should be modified as well by solving equations (18)–(19) at the centre wavelength λ_0_.

From the above discussion, most of the parameters in the optical system could be determined, while among them *d* and *M*_c_ are special variables. In the next section, we will introduce a scheme to explore the desirable values of *d* and *M*_c_ to optimize the system design.

## 3. System optimization   

### 3.1. Spot diagram and spectral distribution quality   

In a system with pre-focusing (diverging) mirror and VLS grating, the optical aberration distribution is more complicated and difficult to calculate precisely. Even implementing the VLS grating, the perfect aberration compensation is difficult to achieve, so the residual aberration terms would spread the spectral line width to reduce the resolving power of the system.

According to the discussion in the previous sections (refer to *A*_4_ in Fig. 2 ▸), we find out:

(*a*) The resolving power decreases with |*M*_c_| (magnification of the pre-convex mirror) monotonously for all spacing values of *d*, while too small |*M*_c_| should be eliminated in the design since the corresponding aberrations would be too large to compensate.

(*b*) The system prefers a larger *d* to deliver a relatively higher resolving power. The larger the value of *d*, the further the pre-convex mirror is separated from the grating, leading to a larger illuminated area on it. As a result, advanced grating manufacturing techniques are needed to enhance the effective optical area with considerably small fabrication errors.

Keeping these in mind, a resolution-enhanced spectrometer could be developed via implementing a pre-divergent mirror, and the system optimization should at least minimize optical aberrations to maintain a decent spectral distribution. In order to evaluate the spectral distribution of the system for different parameter sets, we refer to a ray-tracing program and analyze the spot diagram on the detector plane. The ratio of standard deviation of the meridional coordinates (*y*_*i*_) of the outgoing rays and the line width of the diffracted beam distributed at the detector is used to calibrate the imaging quality at each specific wavelength,
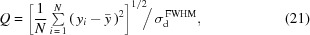
where 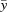 is the average value of *y*_*i*_, *N* is the total number of diffracted rays in the simulation (here it is set to 10000), and the denominator of equation (21) represents the ideal line width of the beam footprint on the detector, and could be calculated by (Li & Li, 2018 ▸)
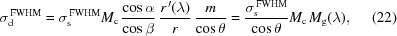
where θ is defined as the angle in between the central diffraction beam and the normal of the X-ray detector, *r* and *r*′(λ) are the object and image distances of the grating, respectively, and 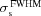 and *M*_g_(λ) are the primary source size and effective magnification of the grating defined in equation (5), respectively.

**Figure 3:**
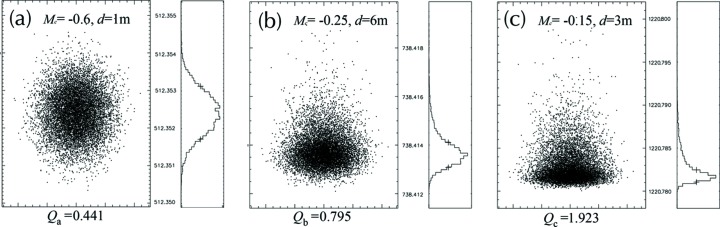
Comparison of the spectral distributions of the system at the detector domain, for three different sets of *d* and *M*_c_, at the radiation wavelength of 5 nm. The imaging quality is evaluated by the justified standard deviation of the meridional coordinates *Q* of the outgoing rays (vertical distribution). The larger the *Q* value, the worse the imaging quality; and vice versa. (*a*) *M*_c_ = −0.6, *d* = 1 m, *Q*_*a*_ = 0.441. (*b*) *M*_c_ = −0.25, *d* = 6 m, *Q*_*b*_ = 0.795. (*c*) *M*_c_ = −0.15, *d* = 3 m, *Q*_*c*_ ≃ 1.923.

Generally, the larger the value of *Q*, the greater the optical dispersion and the worse the imaging quality; and vice versa. The spot diagrams at 5 nm for three different sets of *d* and *M*_c_ were obtained from the *SHADOW* ray-tracing program (Sanchez del Rio *et al.*, 2011 ▸) and presented in Fig. 3 ▸ for comparison, where the *Q* value for each case was calculated to evaluate the corresponding spectral imaging quality.

As depicted in Fig. 3 ▸, the imaging quality of (*a*) is quite good, exhibiting an evenly distributed and symmetric feature, while the image qualities of (*b*) and (*c*) are a lot worse; where the distribution of the outgoing beam deviates from an ideal Gaussian peak, showing certain degrees of asymmetry. The *Q* values of the latter two (*Q*_*b*_ = 0.795, *Q*_c_ ≃ 1.923) are much larger than for the first one (*Q*_*a*_ = 0.441), implicating that the system is not always optimized, especially when the optical aberrations are not well corrected. Generally, the actual resolving power is significantly less than the ideal case, so we establish the criteria *Q* to identify the realistic spectral quality for various cases. However, the parameters of *M*_c_ and *d* are dependent on each other, and searching for an optimal set of parameters is not straightforward. Thus, a machine-learning scheme is introduced to narrow down the pool for exploring the various variables in demand and to improve the efficiency for identification of the optimal system, which will be discussed next.

### 3.2. System optimization through machine-learning scheme   

Following the previous section, the machine-learning scheme is organized as follows: *d* and *M*_c_ are set as the input variables, the rest of parameters of the optical system are either fixed or determined according to the input variables associatively, while the imaging quality *Q* is the output. Through iterative modelling and learning, the machine could nicely predict the imaging quality of the system with different sets of parameters, thus approaching the best values of *d* and *M*_c_.

More specifically, the Support Vector Machine (SVM) is introduced to do the job through implementing the structural risk minimization inductive principle to obtain generalization from a limited number of learning patterns to predict further results (Vapnik, 1963 ▸; Vapnik & Chervonenkis, 1964 ▸). SVM has two main categories: Support Vector Classification (SVC) and Support Vector Regression (SVR) (Vapnik, 2001 ▸); here the latter is utilized to minimize the system errors to achieve generalized performance, where the computation is based on a linear regression function in a multi-dimensional space (≫3) while the input data are mapped via a nonlinear scheme. In current research, we adopted the powerful software *LIBSVM* and model developed by Chang & Lin (2011 ▸).

**Figure 4:**
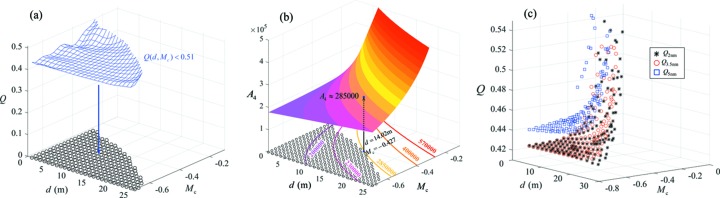
*Q*-value representing the justified standard deviation of the meridional coordinates of the ray-tracing spot diagram, and the ideal resolving power of *A*_4_ is given by equation (4); both of them are crucial parameters for the system optimization. (*a*) *Q* distribution profile (blue mesh, for 5 nm), with dependence on *d* and *M*_c_. The restriction *Q*(*d*,*M*_c_) < 0.51 gives the projection domain in the plane of *Q* = 0, where the small circles on the plane indicate that only within this regime is the quality of the spectral distribution good enough. (*b*) *A*_4_ distribution profile, and the dependence with *d* and *M*_c_. The curves with various colours in the plane of *A*_4_ = 0 are associated with the equal-resolution contour from the *A*_4_ profile. Thus, a set of optimal parameters were found: *M*_c_ = −0.427, *d* = 14.02 m, corresponding to an ideal resolving power of 285000. The scatter plots in (*c*) show *Q* distributions (0.41 < *Q* < 0.55) for the systems with different sets of *M*_c_ and *d* at various wavelengths: 2 nm (stars), 3.5 nm (circles) and 5 nm (squares). Confining *Q*_5nm_ below a certain value (*Q*_5nm_ < 0.51) means that the imaging quality of the entire spectral range is satisfied.

Again, the parameters described at the beginning of Section 2 were used: wavelength range, 2–5 nm; size of light source, 50 µm (r.m.s); beam divergence angle, 20 µrad (r.m.s); Gaussian type; *D*_0_ = 24000 lines cm^−1^, both the incident angles for grating and convex mirror set to 89°, *L* = 30 m *etc*. Multiple sets of *d* and *M*_c_ were used as the two input variables of the support vector machine for training. Besides the preset parameters for each set, the rest of the parameters of the spectrometer, *e.g.* VLS coefficients, radii of mirrors *etc*., could be determined associatively to achieve the system optimization. Then the spectral distribution and image quality were evaluated by the ray-tracing spot diagram and the justified standard deviation *Q* [defined by equation (21)]. There are 233 sets of 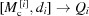 samples generated in total, within certain restrictions (given below), where *i* is the index of the samples; among them, the first 200 samples selected randomly were input to *LIBSVM* for training and calibration, and the last 33 were used as verification. For a system with only two featured input variables, *LIBSVM* can easily gain convergence. An equation of *Q*(*d*,*M*_c_) could be obtained to predict the spectral image quality to reconstruct an optimal system specifically, thus various input quantities of *M*_c_ and *d* would lead to different *Q* values. Then the optimal set possessing the highest ideal resolving power while satisfying the *Q* constraint could be identified. The general restrictions for the system optimization are described below,
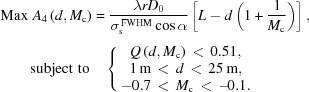
Using a simple grid searching scheme, the best set of parameters were found: *M*_c_ = −0.427, *d* = 14.02 m. The optimization process is demonstrated in Fig. 4 ▸. The blue mesh in Fig. 4(*a*) ▸ shows the *Q* distribution profile with dependence on *d* and *M*_c_, and the regime for *Q*(*d*,*M*_c_) < 0.51 (empirical value) meets the restriction for system optimization. By projecting it onto the plane *Q* = 0, the effective domain for valid *d* and *M*_c_ is determined. When |*M*_c_| is small (|*M*_c_| < 0.3), the optical elements spacing *d* also needs to be small to meet the constraint. On the other hand, when |*M*_c_| is relatively larger, the choices of ‘*d*’ are more flexible. The distribution profile of *A*_4_(*d*, *M*_c_) is plotted in Fig. 4(*b*) ▸; there is a trend of higher resolving power for smaller |*M*_c_| and larger *d*. The colour curves in the plane of *A*_4_ = 0 are associated with the equal-resolution contour from the *A*_4_ profile, *i.e.* casting all available sets of *d* and *M*_c_ with identical ideal resolving power. Meanwhile the valid domain obtained from Fig. 4(*a*) ▸ is plotted on the plane of *A*_4_ = 0 against various contour lines of *A*_4_. It is not difficult to find out that the optimization approaches the contour line with a resolving power of 285000, which intersects with the effective domain to identify an optimal set of parameters: *M*_c_ = −0.427, *d* = 14.02 m. The other parameters of the system were determined associatively and are listed in Table 1 ▸.

It should be pointed out that the results above were obtained by machine learning for the quality of spectral distribution (*Q* function) at 5 nm. Similarly, the machine-learning scheme could be applied to the other wavelengths in the spectral range. Fig. 4(*c*) ▸ demonstrates the *Q* distribution with different sets of *M*_c_ and *d* at wavelengths of 2 nm, 3.5 nm and 5 nm. The vertical axial range is set to 0.41 < *Q* < 0.55, as the ‘zoom-in’ feature of Fig. 4(*a*) ▸ to highlight and compare the magnitudes of the *Q* values for the optimized system at different wavelengths. It can be seen that, within the effective domain (for system optimization), *Q*_2nm_ (black stars) and *Q*_3.5nm_ (red circles, central wavelength) have similar distribution profiles, while *Q*_5nm_ (blue squares) are slightly larger than the other two, implicating that the image quality for 5 nm is lowest throughout the wavelength range. This indicates that optimization of *Q*_5nm_ is not just achieving an optimal system at the single wavelength of 5 nm; the process would lead to an optimal system spanning the entire ‘water window’, *i.e.* 2–5 nm.

## 4. More comments on ray-tracing – aberration and fabrication errors   

In the previous section, we formulated a novel scheme for the design of a resolution-enhanced spectrometer, by implementing a pre-convex mirror to generate a reduced virtual image, which acts as an effectively real source for the VLS grating downstream. The aberrations of the convex mirror should also be considered and combined with the grating in system design and optimization. The SVM is used to explore the optimal parameters more efficiently, and to eliminate the system’s primary aberrations throughout the wavelength range to achieve extremely high resolving power with excellent spectral distribution simultaneously.

In order to evaluate the actual resolving power of a realistic spectrometer *A*_4_, a number of primary factors need to be considered and analysed. First, the spectral line width at the detector due to the light source size is (*i.e.* the ideal line width) (Li & Li, 2018 ▸)
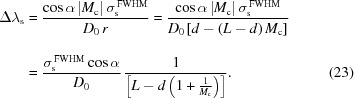
Thus, the ideal spectral resolution could be calculated by *A*_ideal_ = λ/Δλ, assuming a Gaussian beam in an aberration-free optical system, whose resolving power is mainly limited by the light source size 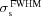, enhanced by a factor of 1/|*M*_c_| from *A*_1_. In a real optical system, the optical aberrations are non-negligible, which will broaden the spectral width distribution of an ideal Gaussian beam substantially, according to
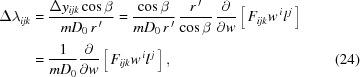
where Δ*y*_*ijk*_ is the meridional beam size at the detector [refer to equation (20)], and the first few dominant aberration terms are (only for the meridional components, thus the sagittal index *l* = 0)
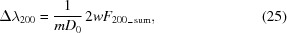


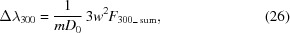


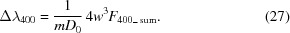
The explicit expressions of *F*_200_sum_, *F*_300_sum_ and *F*_400_sum_ were already given in equations (17)–(19), which are independent of either *w* or *l*.

For an optical system aiming for exceptionally high spectral resolution, the requirements for the fabrication error (or height error) are very critical, including the slope error and surface roughness *etc*. for both the pre-convex mirror and the grating, which broadens the spectral line width by
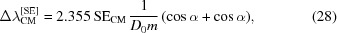


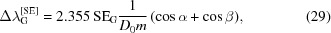
where SE_CM_ and SE_G_ represent the meridional slope error of the convex mirror and grating, respectively. Assuming that they have an identical value, *i.e.* SE_CM_ = SE_G_, then the accumulative slope error of the system is
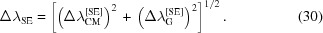
The upper bound of the spectral width due to the slope error [refer to equations (28)–(30)] could be set to that of the source size [refer to equation (23)], then the slope error of the optical element should be
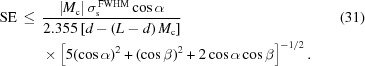
Using the source size and diffraction angle β at 5 nm, the expected slope error should be smaller than 0.1 µrad. Currently the fabrication requirement for SE = 0.1 µrad is very challenging and rare, even for the most advanced grating manufacturing techniques [there are reports about achieving an optical slope error of better than 0.05 µrad though (Dvorak *et al.*, 2016 ▸)]. Since our ultimate goal is to develop a broad-band spectrometer with exceptional resolution over the whole spectral range (>100000), it is worthwhile demanding cutting-edge grating fabrication technology.

**Figure 5:**
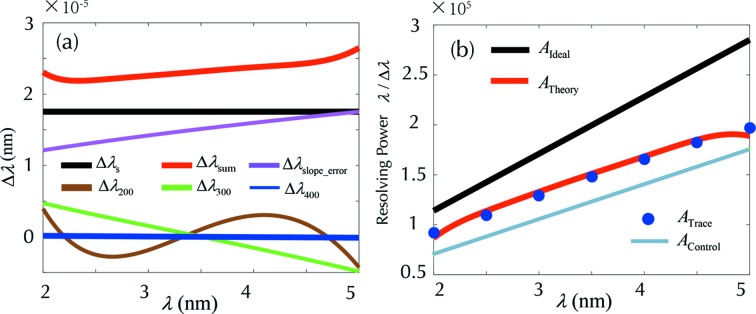
(*a*) The calculated results of the major factors which influence the resolving power of the spectrometer, including the source size (black), the optical fabrication error (purple), the optical aberrations – defocus (brown), coma (green), spherical aberration (blue) and the overall (thick red). The corresponding resolving powers of (*a*) are calculated and presented in (*b*), where the three different types of spectral resolutions are: *A*_ideal_ = λ/Δλ_s_ (black), *A*_theory_ = λ/Δλ_sum_ (red), *A*_trace_ (dot-blue) obtained from the ray-tracing program; and a control signal *A*_control_ (grey) is plotted in the same spectral range, calculated by equation (1), *i.e.* ideal resolution of *A*_1_ with identical *L*.

When all effects in a realistic spectrometer are included, the resolution can be re-calculated,
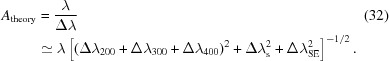
The spectrometer model in Table 1 ▸ could be used to calculate the various spectral distribution terms via implementing equations (23), (25)–(27) and (28)–(30), and the results are shown in Fig. 5(*a*) ▸. The source size term Δλ_s_ seems to be dominating, almost constant within the spectral range (since the source size is assumed to be constant throughout the spectral range). The slope-error term Δλ_SE_ is the second largest component. The spectral broadenings due to three primary aberration components (Δλ_200_, Δλ_300_ or Δλ_400_) are relatively small and well confined.

The corresponding resolving powers for various terms in Fig. 5(*a*) ▸ are exhibited in Fig. 5(*b*) ▸, where the ideal spectral resolution *A*_ideal_ = λ/Δλ_s_ (thick black), the theoretical resolution (thick red) *A*_theory_ = λ/Δλ_sum_, and the result from the ray-tracing program *A*_trace_ (discrete blue disks) and a control group *A*_control_ (grey) calculated by equation (1) using an identical *L*, are overlaid for comparison. Obviously, the theoretical resolving power of a realistic spectrometer *A*_4_ (thick red), including the contribution from slope error and optical aberrations, is still considerably larger than the ideal resolving power of a single-grating spectrometer *A*_1_ (grey). This indicates that, if the precision of grating manufacturing were pushed to the extreme limit, the system would achieve even higher spectral resolution, approaching the ideal value *A*_ideal_ (black).

**Figure 6:**
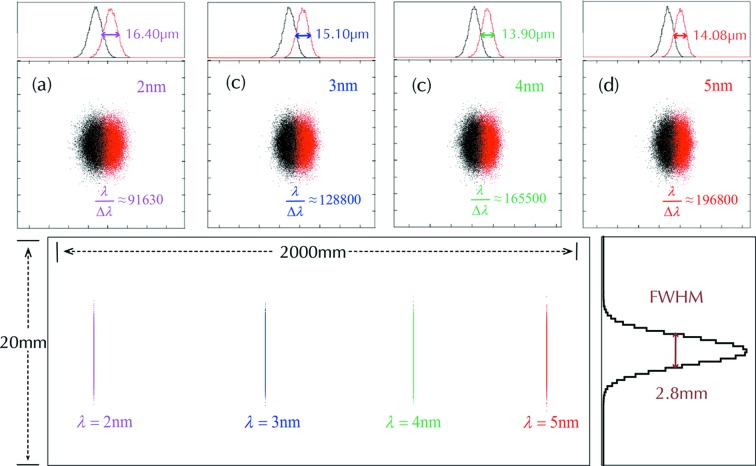
Ray-tracing results for the spectrometer configuration in Table 1 ▸. The spectral profile distributions at the optimal detector plane for the full wavelength range (2–5 nm) are demonstrated at the lower part of the figure, where the detector needs to scan to cover an area of 2000 mm (meridional) × 20 mm (sagittal). The ray-tracing results for each wavelength of 2 nm, 3 nm, 4 nm and 5 nm are presented in (*a*)–(*d*), spanning an identical ‘detector domain’ of 20 mm (vertical) × 0.1 mm (horizontal) for each: the meridional size of the spectrograph is 13–17 µm (FWHM), the sagittal size is about 2.8 mm (FWHM). Then the resolution power at various wavelengths are presented: (*a*) 91630 at 2 nm, (*b*) 128800 at 3 nm, (*c*) 165500 at 4 nm and (*d*) 196800 at 5 nm.

Additionally, the ray-tracing results for the spectrometer with configuration in Table 1 ▸ are presented in Fig. 6 ▸. The bottom part of the figure shows the spectral distributions at the optimal detector plane throughout the ‘water-window’ range (*i.e.* 2–5 nm), where the length scales in the meridional (2000 mm) and sagittal (20 mm) directions are quite different. Figs. 6(*a*)–6(*d*) ▸ exhibit the spectral distribution and resolution at each individual wavelength (2, 3, 4 and 5 nm in terms of λ and λ + Δλ), each in an identical detector domain of a rectangle of dimensions 20 mm (sagittal) × 0.1 mm (meridional). In particular, the FWHM beam widths for each wavelength in the meridional coordinate are illustrated in specific sub-plots, which are set to be larger than the typical pixel size of a CCD detector, ∼10 µm, to guarantee the realization of the spectral resolution. According to equation (6), the image distance of the grating *r*′ should be at least about 30 m for an optimal spectrometer *A*_4_ to achieve the ideal resolving power of 300000. This means that the length scale of the outgoing beam of the spectrometer would be very large, and hence so would the detector range. While our design delivers an excellent flat-field crossing throughout the spectral range, the CCD detector could be mounted and scanned on a more or less straight guide-rail to cover the entire spectrum.

## 5. Discussion and conclusion   

In summary, we report a novel spectrometer design in combination with a cylindrically convex pre-mirror and a cylindrically concave VLS grating (both in the meridional). The design could not only provide a decent flat-field at the detector domain but also enhance the resolving power substantially. Our main findings in the current research are: (1) If a convex mirror is inserted in between the light source and the grating to create a reduced virtual image (acting as a secondary real source point for the grating), the resolution of the system would be enhanced. (2) Generally, if a pre-mirror (convex or concave) is inserted upstream of the grating, its optical aberration should be included and justified (*e.g.* the magnification, creating a real or virtual image), in order to calculate and compensate the overall aberration of the system accurately. (3) A realistic optical system always possesses errors, *e.g.* optical aberrations and fabrication errors, thus the beam spectral distribution would be broader than and deviate from an aberration-free ideal Gaussian distribution; and the standard deviation of the outgoing beam’s spot diagram could be used to reflect the image quality. (4) The support vector machines can quickly learn from the input data and reconstruct the prediction formula to explore the optimal system with excellent imaging quality. By implementing a nonlinear programming script, an optimized parameter set of *M*_c_ and *d*, associated with the highest resolving power, could be identified. (5) A spectrometer system with extremely high resolving power always has very high demands for precise manufacturing of optical components, *i.e.* requiring exceptionally small slope errors and surface roughness for the optical elements in the system.

The position and magnification of the pre-convex mirror are the crucial parameters in the current spectrometer design, which also constrain the selection for the object and image distances of the grating, thus reducing the number of variables for system optimization. Implementation of a machine-learning scheme could explore and identify the optimal system delivering an excellent resolution while maintaining minimal optical aberrations with fairly high efficiency. In general, by implementing the SVM in a single PC with a four-core CPU, it would take roughly an hour to explore and establish an optimal system with appropriate parameters. Although we mainly discussed a spectrometer design for the ‘water window’, the algorithm owns universal adaptability, which could be easily extended to a much broader photon energy range through an appropriate modification of the design parameters. We are planning to utilize the current scheme to develop a high-resolution spectrometer spanning the ∼keV range in the near future. It is worthwhile mentioning that the scheme could be applied straightforwardly to many types of experiments which pursue highest spectral resolution through the introduction of the resolving power enhancement structure to grating diffraction-based instruments. It could provide a relatively higher resolving power compared with a single-grating spectrometer (assuming both systems possess an identical primary object distance of *L*, obviously). More remarkably, in the current spectrometer design, the extremely high resolving power (100000–200000) could be realized at a rather large source size (50 µm r.m.s.), which is not possible for any type of previous designs.

## Figures and Tables

**Table 1:** Design parameters of the optimized spectrometer (*A*_4_)

Source	
Type	Gaussian
Size	50 µm (r.m.s.)
Divergence angle	20 µrad (r.m.s.)
*L*	30 m

Convex mirror	
α_c_	89°
*r*_c_	15.98 m
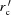	−6.82 m
*d*	14.02 m
*M*_c_	−0.427
*R*_c_	−1364.70 m

Concave VLSG	
α	89°
*r*	20.84 m
*r*′	31 m
*R*	2706.50 m
VLS coefficient	*D*_0_ = 24000 lines cm^−1^
	*D*_1_ = 14.55 lines cm^−2^
	*D*_2_ = 0.0073 lines cm^−3^
	*D*_3_ = 3.689 × 10^−6^ lines cm^−4^

Footprint (FWHM) on the convex mirror surface, *w*_CM_	3.97 cm
Footprint (FWHM) on the grating surface, *w*_G_	12.14 cm

Required slope error (meridional)	
SE_CM_	<0.1 µrad
SE_G_	<0.1 µrad
